# A pilot placebo controlled randomised trial of glyceryl trinitrate for retained placenta (GOTIT trial): assessing the potential for progressing to a full RCT

**DOI:** 10.1186/1745-6215-16-S2-P19

**Published:** 2015-11-16

**Authors:** Susan Morrow, Julia Lawton, Nina Hallowell, Gladys McPherson, John Norrie, Fiona Denison

**Affiliations:** 1 University of Edinburgh, Edinburgh, UK; 2 University of Aberdeen, Aberdeen, UK

## Background

The aim of the NIHR funded GOT-IT trial is to determine the clinical and cost effectiveness of a non-surgical management for retained placenta; sublingual GTN spray. We report the results of a UK based internal pilot randomised controlled trial with embedded qualitative research to test the suitability of trial processes and determine achievable recruitment rates.

## Methods

Eight sites were selected to take part in the pilot study. To facilitate sufficient suitably trained staff being available 24/7 to recruit, a trial specific GCP training package was developed. Qualitative interviews were undertaken with trial participants and clinical staff to explore barriers and facilitators to recruitment. The target recruitment for the internal pilot was 78 participants by the end of May 2015.

## Results

Pilot sites were given the green light for recruitment between October 2014 and January 2015. The first participant was recruited on 13 October 2014. By February 2015 all sites had recruited at least one participant. Seventy eight participants were recruited ahead of target on 13 April 2015. Six sites used the trial specific GCP training. It was generally well received, facilitating earlier trial start-up and better recruitment rates compared to sites using traditional GCP training packages. The major themes from qualitative interviews support the finding that recruitment is likely to go well in the main trial.

## Conclusions

Results from the qualitative study indicate that a full-scale trial is feasible and likely to recruit well. A trial specific GCP training package facilitates earlier start up and achievable recruitment rates.

